# *N* ^1^,*N* ^2^-Bis(6-methyl-2-pyrid­yl)formamidine

**DOI:** 10.1107/S1600536809015591

**Published:** 2009-04-30

**Authors:** Chia-Jun Wu, Chang-Wei Su, Chun-Wei Yeh, Jhy-Der Chen, Ju-Chun Wang

**Affiliations:** a Department of Chemistry, Chung-Yuan Christian University, Chung-Li, Taiwan, Republic of China; b Department of Chemistry, Soochow University, Taipei, Taiwan, Republic of China

## Abstract

In the crystal structure of the title mol­ecule, C_13_H_14_N_4_, the two pyridyl rings are not coplanar but twisted about the C—N bond with an inter­planar angle of 71.1 (1)°. In the crystal, the mol­ecules form dimers, situated on crystallographic centres of inversion, which are connected *via* a pair of N—H⋯N hydrogen bonds. C—H⋯π-electron ring inter­actions are also present in the crystal structure. The title mol­ecule adopts an *s–cis–anti–s–cis* conformation in the solid state.

## Related literature

For related structures, see: Wu *et al.* (2009 ▶); Liang *et al.* (2003 ▶); Yang *et al.* (2000 ▶); Radak *et al.* (2001 ▶). For the synthesis, see: Roberts (1949 ▶).
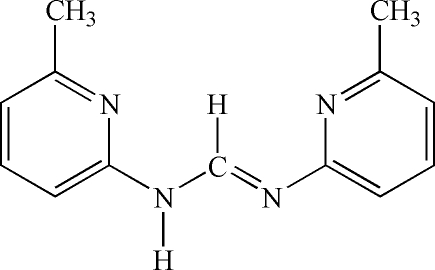


## Experimental

### 

#### Crystal data

C_13_H_14_N_4_
*M* *_r_* = 226.28
Monoclinic, 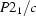

*a* = 6.0364 (4) Å
*b* = 19.6697 (14) Å
*c* = 10.4040 (7) Å
β = 96.081 (1)°
*V* = 1228.36 (15) Å^3^
*Z* = 4
Mo *K*α radiation
μ = 0.08 mm^−1^
*T* = 298 K
0.5 × 0.5 × 0.3 mm

#### Data collection

Bruker SMART 1000 diffractometer
Absorption correction: multi-scan (**SADABS**; Bruker, 1997 ▶) *T*_min_ = 0.683, *T*_max_ = 0.792 (expected range = 0.842–0.977)
7002 measured reflections
2912 independent reflections
2313 reflections with *I* > 2σ(*I*)
*R*_int_ = 0.110

#### Refinement

*R*[*F* ^2^ > 2σ(*F* ^2^)] = 0.060
*wR*(*F* ^2^) = 0.148
*S* = 1.09
2912 reflections
161 parameters
H atoms treated by a mixture of independent and constrained refinement
Δρ_max_ = 0.16 e Å^−3^
Δρ_min_ = −0.21 e Å^−3^

### 

Data collection: *SMART* (Bruker, 1997 ▶); cell refinement: *SAINT* (Bruker, 1997 ▶); data reduction: *SAINT* and *SHELXTL* (Sheldrick, 2008 ▶); program(s) used to solve structure: *SHELXS97* (Sheldrick, 2008 ▶); program(s) used to refine structure: *SHELXL97* (Sheldrick, 2008 ▶); molecular graphics: *SHELXTL*; software used to prepare material for publication: *SHELXTL*.

## Supplementary Material

Crystal structure: contains datablocks I, global. DOI: 10.1107/S1600536809015591/fb2144sup1.cif

Structure factors: contains datablocks I. DOI: 10.1107/S1600536809015591/fb2144Isup2.hkl

Additional supplementary materials:  crystallographic information; 3D view; checkCIF report

## Figures and Tables

**Table 1:** Hydrogen-bond geometry (Å, °)

*D*—H⋯*A*	*D*—H	H⋯*A*	*D*⋯*A*	*D*—H⋯*A*
N3—H3*N*⋯N2^i^	0.89 (2)	2.09 (2)	2.9775 (19)	173 (2)
C1—H1*B*⋯*Cg*1^ii^	0.96	2.83	3.644 (2)	143
C11—H11*A*⋯*Cg*1^iii^	0.93	2.96	3.757 (2)	145

Symmetry codes: (i) 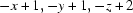; (ii) 
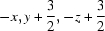
; (iii) 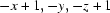. *Cg*1 is the centroid of the N1,C2–C6 ring.

**Fig. 1.**
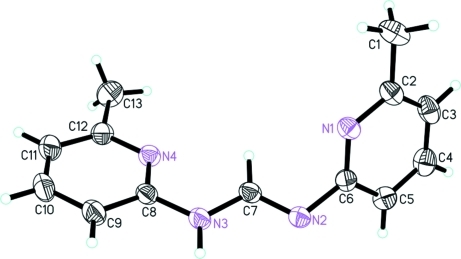
The title molecule with the labelling scheme. The displacement ellipsoids are drawn at the 30% probability level.

**Fig. 2.**
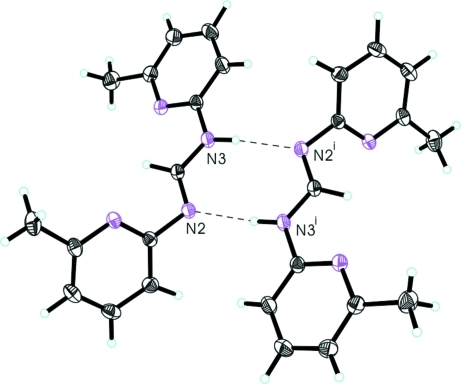
View on the dimers bind by the hydrogen bonds, which are shown as dashed lines. Symmetry code: (i) -x+1, -y+1, -z+2.

## supplementary crystallographic information

### Comment

The title molecule as well as its anion have been used as bridging ligands in the coordination chemistry (Liang *et al.*, 2003; Yang *et al.*, 2000; Radak *et al.*, 2001). In the present work, the structure of the title molecule (Fig. 1) has been determined to explore its ligand conformation.

The molecules form dimers that are interconnected *via* a pair of N—H···N hydrogen bonds (Tab. 1, Fig. 2). Moreover, there are also C—H···π-electron ring interactions (Tab. 1) in the structure. The conformation in the title molecule in the structure is *s-cis-anti-s-cis*. This conformation is in contrast to that one found in *N^1^,N^2^*-bis(2-pyridyl)formamidine, which is *s-trans-syn-s-cis* (Wu *et al. *, 2009).

### Experimental

The title compound was prepared according to the procedure described by Roberts (1949). 2-Aminopyridine (12.96 g, 0.12 mol) and triethyl orthoformate (11.8 g, 0.06 mol) were placed under nitrogen into a flask. The mixture was then refluxed for 8 h to give a brown solid. Dichloromethane (10 ml) was then added to dissolve the solid and then hexane (25 ml) was added to induce the precipitation. The precipitate was filtered and dried under vacuum to give a light yellow solid with a yield of 83%. By dissolving the solid in dichloromethane, followed by allowing the solution to evaporate slowly under air, several yellow crystals suitable for X-ray crystallography were obtained. One block crystal with size of 0.5 x 0.5 x 0.3 mm was used for data collection.

### Refinement

All the hydrogen atoms were discernible in the difference Fourier maps. However, they were situated into the idealized positions and constrained by the riding atom approximation: *C*—H_methyl_ = 0.96 Å while the methyls were allowed to rotate about their respective axes; *C*—H_aryl_ = 0.93 Å; *U*_iso_(H_methyl_) = 1.5*U*_eq_(C_methyl_); *U*_iso_(H_aryl_) = 1.2*U*_eq_(C_aryl_). The amine hydrogen atom (H3N) that is involved in the N-H···N hydrogen bond was freely refined.

### Crystal data

**Table:**

C_13_H_14_N_4_	*F*(000) = 480
*M**_r_* = 226.28	*D*_x_ = 1.224 Mg m^−^^3^
Monoclinic, *P*2_1_/*c*	Mo *K*α radiation, λ = 0.71073 Å
Hall symbol: -P 2ybc	Cell parameters from 7002 reflections
*a* = 6.0364 (4) Å	θ = 2.1–28.3°
*b* = 19.6697 (14) Å	µ = 0.08 mm^−^^1^
*c* = 10.4040 (7) Å	*T* = 298 K
β = 96.081 (1)°	Block, yellow
*V* = 1228.36 (15) Å^3^	0.5 × 0.5 × 0.3 mm
*Z* = 4	

### Data collection

**Table:**

Bruker SMART 1000 diffractometer	2912 independent reflections
Radiation source: fine-focus sealed tube	2313 reflections with *I* > 2σ(*I*)
graphite	*R*_int_ = 0.110
φ and ω scans	θ_max_ = 28.3°, θ_min_ = 2.1°
Absorption correction: multi-scan (*SADABS*; Bruker, 1997)	*h* = −8→8
*T*_min_ = 0.683, *T*_max_ = 0.792	*k* = −22→26
7002 measured reflections	*l* = −13→8

### Refinement

**Table:**

Refinement on *F*^2^	Secondary atom site location: difference Fourier map
Least-squares matrix: full	Hydrogen site location: difference Fourier map
*R*[*F*^2^ > 2σ(*F*^2^)] = 0.060	H atoms treated by a mixture of independent and constrained refinement
*wR*(*F*^2^) = 0.148	*w* = 1/[σ^2^(*F*_o_^2^) + (0.0514*P*)^2^ + 0.2913*P*] where *P* = (*F*_o_^2^ + 2*F*_c_^2^)/3
*S* = 1.09	(Δ/σ)_max_ < 0.001
2912 reflections	Δρ_max_ = 0.16 e Å^−^^3^
161 parameters	Δρ_min_ = −0.21 e Å^−^^3^
0 restraints	Extinction correction: *SHELXL97* (Sheldrick, 2008), Fc^*^=kFc[1+0.001xFc^2^λ^3^/sin(2θ)]^-1/4^
50 constraints	Extinction coefficient: 0.050 (6)
Primary atom site location: structure-invariant direct methods	

### Special details

**Table:**

Geometry. All e.s.d.'s (except the e.s.d. in the dihedral angle between two l.s. planes) are estimated using the full covariance matrix. The cell e.s.d.'s are taken into account individually in the estimation of e.s.d.'s in distances, angles and torsion angles; correlations between e.s.d.'s in cell parameters are only used when they are defined by crystal symmetry. An approximate (isotropic) treatment of cell e.s.d.'s is used for estimating e.s.d.'s involving l.s. planes.
Refinement. Refinement of *F*^2^ against ALL reflections. The weighted *R*-factor *wR* and goodness of fit *S* are based on *F*^2^, conventional *R*-factors *R* are based on *F*, with *F* set to zero for negative *F*^2^. The threshold expression of *F*^2^ > σ(*F*^2^) is used only for calculating *R*-factors(gt) *etc*. and is not relevant to the choice of reflections for refinement. *R*-factors based on *F*^2^ are statistically about twice as large as those based on *F*, and *R*- factors based on ALL data will be even larger.

### Fractional atomic coordinates and isotropic or equivalent isotropic displacement parameters (Å^2^)

**Table:**

	*x*	*y*	*z*	*U*_iso_*/*U*_eq_	
N1	0.1831 (2)	0.44487 (7)	0.66067 (13)	0.0459 (3)	
N2	0.2674 (2)	0.49243 (7)	0.86759 (12)	0.0470 (3)	
N3	0.5357 (2)	0.57705 (7)	0.89283 (13)	0.0497 (4)	
N4	0.4953 (2)	0.67233 (7)	0.76126 (13)	0.0481 (3)	
C1	0.1286 (4)	0.39836 (12)	0.44484 (19)	0.0733 (6)	
H1B	0.1775	0.4392	0.4059	0.110*	
H1C	0.0140	0.3772	0.3874	0.110*	
H1D	0.2521	0.3677	0.4613	0.110*	
C2	0.0383 (3)	0.41552 (8)	0.56991 (16)	0.0514 (4)	
C3	−0.1794 (3)	0.40215 (10)	0.5912 (2)	0.0620 (5)	
H3A	−0.2780	0.3832	0.5262	0.074*	
C4	−0.2488 (3)	0.41725 (10)	0.7103 (2)	0.0640 (5)	
H4B	−0.3947	0.4083	0.7263	0.077*	
C5	−0.1007 (3)	0.44564 (9)	0.80515 (18)	0.0538 (4)	
H5A	−0.1423	0.4549	0.8869	0.065*	
C6	0.1131 (3)	0.46006 (8)	0.77451 (15)	0.0433 (3)	
C7	0.3682 (3)	0.54351 (8)	0.82345 (15)	0.0455 (4)	
H7A	0.3238	0.5581	0.7396	0.055*	
C8	0.6236 (3)	0.63789 (8)	0.85033 (15)	0.0457 (4)	
C9	0.8327 (3)	0.65966 (10)	0.90180 (19)	0.0627 (5)	
H9A	0.9187	0.6341	0.9635	0.075*	
C10	0.9085 (4)	0.72061 (12)	0.8581 (2)	0.0751 (6)	
H10A	1.0476	0.7370	0.8908	0.090*	
C11	0.7783 (4)	0.75724 (10)	0.7662 (2)	0.0684 (6)	
H11A	0.8277	0.7986	0.7367	0.082*	
C12	0.5739 (3)	0.73169 (9)	0.71856 (17)	0.0540 (4)	
C13	0.4238 (4)	0.76673 (12)	0.6146 (2)	0.0764 (6)	
H13A	0.2724	0.7640	0.6345	0.115*	
H13B	0.4367	0.7450	0.5330	0.115*	
H13C	0.4666	0.8136	0.6097	0.115*	
H3N	0.602 (3)	0.5592 (11)	0.966 (2)	0.064 (6)*	

### Atomic displacement parameters (Å^2^)

**Table:**

	*U*^11^	*U*^22^	*U*^33^	*U*^12^	*U*^13^	*U*^23^
N1	0.0435 (7)	0.0473 (7)	0.0455 (7)	0.0045 (5)	−0.0024 (5)	−0.0009 (5)
N2	0.0501 (7)	0.0479 (7)	0.0413 (7)	−0.0025 (6)	−0.0021 (5)	0.0012 (5)
N3	0.0584 (8)	0.0448 (7)	0.0428 (7)	−0.0054 (6)	−0.0082 (6)	0.0033 (6)
N4	0.0531 (7)	0.0449 (7)	0.0455 (7)	0.0010 (6)	0.0018 (6)	0.0001 (6)
C1	0.0852 (14)	0.0787 (14)	0.0535 (10)	0.0134 (12)	−0.0042 (10)	−0.0171 (10)
C2	0.0541 (9)	0.0462 (9)	0.0510 (9)	0.0083 (7)	−0.0082 (7)	−0.0049 (7)
C3	0.0515 (9)	0.0568 (10)	0.0727 (12)	−0.0009 (8)	−0.0160 (8)	−0.0071 (9)
C4	0.0404 (8)	0.0656 (12)	0.0847 (14)	−0.0037 (8)	−0.0002 (8)	0.0039 (10)
C5	0.0466 (9)	0.0583 (10)	0.0567 (10)	0.0035 (7)	0.0068 (7)	0.0021 (8)
C6	0.0425 (8)	0.0403 (7)	0.0457 (8)	0.0032 (6)	−0.0020 (6)	0.0031 (6)
C7	0.0513 (9)	0.0441 (8)	0.0394 (7)	0.0019 (7)	−0.0033 (6)	0.0004 (6)
C8	0.0529 (9)	0.0426 (8)	0.0406 (8)	−0.0018 (6)	0.0006 (6)	−0.0041 (6)
C9	0.0642 (11)	0.0625 (11)	0.0569 (10)	−0.0101 (9)	−0.0145 (8)	0.0035 (8)
C10	0.0751 (13)	0.0725 (13)	0.0732 (13)	−0.0292 (11)	−0.0123 (10)	−0.0006 (10)
C11	0.0855 (14)	0.0527 (10)	0.0654 (12)	−0.0207 (10)	0.0010 (10)	0.0008 (9)
C12	0.0693 (11)	0.0423 (8)	0.0506 (9)	−0.0011 (8)	0.0069 (8)	−0.0017 (7)
C13	0.0903 (15)	0.0602 (12)	0.0768 (14)	0.0066 (11)	0.0008 (11)	0.0175 (10)

### Geometric parameters (Å, °)

**Table:**

N1—C6	1.333 (2)	C4—C5	1.378 (3)
N1—C2	1.347 (2)	C4—H4B	0.9300
N2—C7	1.285 (2)	C5—C6	1.391 (2)
N2—C6	1.4212 (19)	C5—H5A	0.9300
N3—C7	1.350 (2)	C7—H7A	0.9300
N3—C8	1.400 (2)	C8—C9	1.386 (2)
N3—H3N	0.90 (2)	C9—C10	1.377 (3)
N4—C8	1.329 (2)	C9—H9A	0.9300
N4—C12	1.353 (2)	C10—C11	1.376 (3)
C1—C2	1.502 (3)	C10—H10A	0.9300
C1—H1B	0.9600	C11—C12	1.375 (3)
C1—H1C	0.9600	C11—H11A	0.9300
C1—H1D	0.9600	C12—C13	1.503 (3)
C2—C3	1.380 (3)	C13—H13A	0.9600
C3—C4	1.382 (3)	C13—H13B	0.9600
C3—H3A	0.9300	C13—H13C	0.9600
			
C6—N1—C2	118.40 (14)	C5—C6—N2	119.49 (15)
C7—N2—C6	114.08 (13)	N2—C7—N3	123.18 (14)
C7—N3—C8	122.43 (14)	N2—C7—H7A	118.4
C7—N3—H3N	120.3 (14)	N3—C7—H7A	118.4
C8—N3—H3N	117.1 (14)	N4—C8—C9	123.49 (16)
C8—N4—C12	118.06 (15)	N4—C8—N3	116.34 (14)
C2—C1—H1B	109.5	C9—C8—N3	120.17 (15)
C2—C1—H1C	109.5	C10—C9—C8	117.52 (18)
H1B—C1—H1C	109.5	C10—C9—H9A	121.2
C2—C1—H1D	109.5	C8—C9—H9A	121.2
H1B—C1—H1D	109.5	C11—C10—C9	120.01 (18)
H1C—C1—H1D	109.5	C11—C10—H10A	120.0
N1—C2—C3	121.81 (16)	C9—C10—H10A	120.0
N1—C2—C1	115.82 (17)	C12—C11—C10	118.93 (18)
C3—C2—C1	122.37 (16)	C12—C11—H11A	120.5
C2—C3—C4	119.16 (16)	C10—C11—H11A	120.5
C2—C3—H3A	120.4	N4—C12—C11	121.97 (17)
C4—C3—H3A	120.4	N4—C12—C13	115.27 (17)
C5—C4—C3	119.60 (17)	C11—C12—C13	122.75 (17)
C5—C4—H4B	120.2	C12—C13—H13A	109.5
C3—C4—H4B	120.2	C12—C13—H13B	109.5
C4—C5—C6	117.76 (17)	H13A—C13—H13B	109.5
C4—C5—H5A	121.1	C12—C13—H13C	109.5
C6—C5—H5A	121.1	H13A—C13—H13C	109.5
N1—C6—C5	123.18 (15)	H13B—C13—H13C	109.5
N1—C6—N2	117.34 (14)		

### Hydrogen-bond geometry (Å, °)

**Table:**

*D*—H···*A*	*D*—H	H···*A*	*D*···*A*	*D*—H···*A*
N3—H3N···N2^i^	0.89 (2)	2.09 (2)	2.9775 (19)	173 (2)
C1—H1B···Cg1^ii^	0.96	2.83	3.644 (2)	143
C11—H11A···Cg1^iii^	0.93	2.96	3.757 (2)	145

Symmetry codes: (i) −*x*+1, −*y*+1, −*z*+2; (ii) −*x*, *y*+3/2, −*z*+3/2; (iii) −*x*+1, −*y*, −*z*+1.
